# Estimating the Accuracy of Anal Cytology in the Presence of an Imperfect Reference Standard

**DOI:** 10.1371/journal.pone.0012284

**Published:** 2010-08-19

**Authors:** William C. Mathews, Edward R. Cachay, Joseph Caperna, Amy Sitapati, Bard Cosman, Ian Abramson

**Affiliations:** 1 Department of Medicine, University of California San Diego, San Diego, California, United States of America; 2 Department of Surgery, University of California San Diego, San Diego, California, United States of America; 3 Department of Mathematics, University of California San Diego, San Diego, California, United States of America; University of Toronto, Canada

## Abstract

**Background:**

The study aim is to estimate sensitivity and specificity of anal cytology for histologic HSIL in analyses adjusted for the imperfect biopsy reference standard.

**Methods and Principal Findings:**

Retrospective cohort study of an anal dysplasia screening program for HIV infected adults. We estimated the prevalence of histologic HSIL by concurrent cytology category and the associated cytology ROC area. Cytology operating characteristics for HSIL were estimated and adjusted for the imperfect reference standard by 3 methodologies. The study cohort included 261 patients with 3 available measures: (1) referral cytology; (2) HRA cytology; and (3) HRA directed biopsy. The prevalence of biopsy HSIL varied according to the concurrent HRA cytology result: 64.5% for HSIL or ASC-H, 12.6% for LSIL, 10.9% for ASCUS, and 6.3% for no abnormality. The cytology ROC area was 0.78. The observed prevalence of HSIL was 37% (referral cytology), 24% (HRA cytology), and 24% (HRA biopsy). Unadjusted estimates of sensitivity and specificity of cytology were 0.66 and 0.90, respectively. Adjusted estimates varied from 0.47–0.89 (sensitivity) and 0.89—1.0 (specificity).

**Conclusions:**

Analysis of a single dataset yields widely different estimates of anal cytology operating characteristics that depend on difficult to verify assumptions regarding the accuracy of the imperfect reference standard.

## Introduction

In a systematic review dealing with screening HIV-infected individuals for precursors of anal cancer, Chiao et al. reported that, among studies using similar methodology, the operating characteristics of anal cytological examination for high resolution anoscopy (HRA) directed biopsy varied from 69% to 93% (sensitivity) and from 32% to 59% (specificity) [1]. The low reported specificity implies a high false positive rate for cytology. However, this inference is flawed because it assumes that the reference standard, HRA-directed biopsy is a perfect gold standard that results in no misclassification of true disease status. Our study aim was to estimate the sensitivity and specificity of anal cytology for high grade anal dysplasia in analyses adjusted and unadjusted for the imperfect nature of the reference standard (HRA directed anal biopsy).

## Methods

This study was conducted according to the principles expressed in the Declaration of Helsinki. The study was approved by the Institutional Review Board of the University of California at San Diego (UCSD) Human Research Protection Program, project# 071931. All patients provided written informed consent for the collection of samples and subsequent analysis.

In July 2000, the Owen Clinic, the adult HIV clinic at UCSD Medical Center in San Diego, implemented routine screening for anal cancer and its precursors. Clinic protocol advises primary care providers to perform a digital rectal examination and obtain anal cytology on all new patients and thereafter annually. Patients found to have abnormal cytology (any results other than “no atypical or malignant cells”), abnormalities on digital rectal examination, or anorectal symptoms are referred for HRA. Previous publications from the Owen Clinic dysplasia cohort described findings from the initial implementation phase [2] and from a comprehensive evaluation of the screening program through 2005 [3]. The current retrospective cohort analysis includes cytologic and histopathologic results from screening procedures performed between May 2007–October 2009. The analytic data set includes 3 primary variables: (1) the referral cytology (obtained by the primary care provider as the first stage of screening); (2) the HRA cytology (obtained by the HRA operator at the time of high resolution anoscopy); and (3) punch biopsy (obtained using a baby Tischler forceps at time of HRA). Cytology specimens were obtained with a moistened Dacron swab (PurFybr, Munster IN) and processed using the SurePath Liquid-based Pap Test system [4], [5]. High resolution anoscopy was performed by four internists following a previously described protocol [6]. The number of biopsies obtained was operator dependent. For analyses presented here, the most severe histologic category was assigned if multiple biopsies were performed. Both cytologic and histopathologic categories were assigned using the 2001 Bethesda System for reporting cervical cytology [7].

Following presentation of descriptive results, we present the prevalence of biopsy high grade dysplasia according to HRA cytology result and the associated receiver operating characteristic (ROC) [8] area for high grade dysplasia on biopsy as the cytology cut point is varied across 4 result categories: (1)high grade squamous intraepithelial lesion (HSIL) or atypical squamous cells can't rule out high grade (ASC-H); (2) low grade squamous intraepithelial lesion (LSIL); (3) atypical squamous cells of uncertain significance (ASCUS); (4) no atypical or malignant cells (NAMC). We then present estimates of the operating characteristics (sensitivity [SE], specificity [SP], likelihood ratio positive [LRP], and likelihood ratio negative [LRN]) of binary coded HRA cytology (cut point at high grade vs. not high grade) for HSIL on biopsy. Histopathologically, HSIL includes the categories of moderate dysplasia (anal intraepithelial neoplasia 2 [AIN 2]), severe dysplasia (AIN 3) and carcinoma-in-situ (CIS). These operating characteristics are presented first without adjustment, assuming that punch biopsy diagnosis defines true disease status. Recognizing that the yield of both cytology and biopsy are operator dependent, we present the unadjusted sensitivity and specificity separately by anoscopist and also pooled across anoscopists using a random effects meta analytic model implemented in Stata 11.0 (Stata Corporation, College Station, Texas). We then present three adjusted estimates of cytology sensitivity and specificity.

The first adjustment assumes: (1) that anal punch biopsy is itself measured with error and has the same sensitivity and specificity for histopathologic *anal HSIL* as was reported for histopathologic *cervical HSIL* by Byrom et al.[9], who compared cervical punch biopsy to LLETZ (large loop excision of the transformation zone) as a reference standard; and (2) that cytology and punch biopsy are conditionally independent given true disease status [10]. Byrom reported that the sensitivity and specificity of colposcopically directed punch biopsy for high-grade CIN (defined as CIN 2 or CIN 3) was 74% and 91%, respectively [9]. Staquet et al. derived equations (equations 8 (
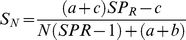
}and 9 (
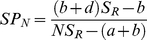
 }) [11] to estimate the sensitivity and specificity of a new test when: (1) the reference standard is imperfect but with known sensitivity and specificity and (2) the new test and the reference standard are conditionally independent given true disease status.

The second adjustment assumes that the punch biopsy has unknown sensitivity but 100% specificity. Although the assumption of 100% specificity for punch biopsy is only approximately true, it could be justified from a clinical perspective in that there is more concern with HRA sampling error (reducing biopsy sensitivity) than with false positive biopsy results. The assumption of 100% specificity amounts to an assumption that punch biopsy yields pathognomonic results. If this assumption is justified, then Staquet has shown that the sensitivity of new test (here anal cytology) is estimated without bias, but that specificity, positive predictive value, and disease prevalence are underestimated while negative predictive value is overestimated. Staquet has shown further that, under this scenario, “the true value of the specificity of the new test is confined between 100% and a lower bound which is the observed specificity (of the new test)” [11]. If equation 19 in Staquet's publication is solved for test specificity, the dependence of test specificity on disease prevalence can be easily explored (see Appendix S1).

The third adjustment is based on *latent class analysis* (LCA), a statistical method for combining test results (here referral cytology, HRA cytology, and HRA biopsy) to estimate the true but unknown disease status (HSIL or not HSIL). This approach has the advantage of taking into account information from multiple sources in estimating true disease status but, in its traditional form, requires a minimum of three conditionally independent tests [10], [12]. LCA is part of a family of statistical models known as *mixture models* and is a categorical data analog of the continuous variable methodology of factor analysis. As with other latent variable techniques, LCA postulates the existence of an error-free latent variable (in our case the presence or absence of HSIL in anal canal tissue) and estimates the value of this latent variable by modeling, commonly through the expectation-maximization (EM) algorithm, observed variables that are themselves subject to measurement error (here anal cytology and HRA directed punch biopsy). Accessible introductions to LCA methodology are available [13], [14]. In utilizing LCA to model sensitivity and specificity of HRA cytology, we imposed the clinically sensible restriction that when the HRA directed biopsy showed HSIL, that diagnosis was considered to be known with certainty. If the biopsy did not show HSIL, the LCA model was allowed to assign a value to the latent disease status based on information from all three indicator variables (referral cytology, HRA cytology, and HRA biopsy). We report the sensitivity and specificity estimates from: (1) the 7-parameter LCA model without relaxation of the conditional independence assumption and (2) the 10-parameter model that relaxes the conditional independence assumption by allowing estimation of direct effects among the three pairs of indicator variables. These LCA models were estimated using Latent Gold 4.5 (Statistical Innovations, Inc., Belmont, MA).

Finally, we present a sensitivity analysis of the impact of the varying estimates of unadjusted and adjusted HRA cytology operating characteristics on the post test probability of anal HSIL disease. This analysis illustrates the range of uncertainty regarding the probability that a patient with a non-HSIL anal cytology result actually harbors HSIL changes if that patient was selected from a population with known HSIL prevalence.

## Results

Between 1 May 2007 and 21 October 2009, 407 HIV infected patients underwent HRA with biopsy. By sex, 93% were male; 42% were non-white. By HIV transmission risk factor, 70% were men having sex with men (MSM) and 13% were injection drug users (not MSM). Their median age (IQR) was 45 (39–51) years. Median (IQR) CD4 and HIV plasma viral load were 429 (261–611) cell/µL and 1.70 (1.68–2.14) log_10_ copies/µL, respectively. Of the 407 who underwent HRA-directed biopsy, 371 (91%) had a concurrent HRA cytology and 261 (64%) had both a referral and HRA cytology available for analysis. For consistency, analyses will be restricted to the 261 patients with complete data on the 3 primary variables (referral cytology, HRA cytology, and HRA directed biopsy). For this set of patients, the observed prevalence of HSIL was 37% (referral cytology), 24% (HRA cytology), and 24% (HRA biopsy). The prevalence of biopsy HSIL varied according to the concurrent HRA cytology result: 64.5% (40/62) for HSIL or ASC-H, 12.6% (13/103) for LSIL, 10.9% (7/64) for ASCUS, and 6.3% (2/32) for NAMC. The receiver operating characteristic (ROC) area for the 4-category coding of cytology results (HSIL or ASC-H, LSIL, ASCUS, NAMC) for HSIL on biopsy was 0.78 (95% CI: 0.72–0.85). The median (IQR) time between referral and HRA cytologies for the 261 patients in the analysis was 158 (66,295) days, with a range of 2 to 839 days. We categorized both cytology and biopsy results into corresponding binary groups as follows: (1) for cytology, (HSIL or ASC-H) vs. (LSIL, ASCUS, atypia, or NAMC); for biopsy, (AIN 2, AIN 3, or CIS) vs. (normal mucosa or AIN 1). Table 1 presents the cross classification of the three binary coded primary analytic variables.

**Table 1:** Cross classification of referral cytology, HRA cytology, and HRA directed biopsy (n = 261 patients).

	Biopsy and HRA cytology				
Referral Cytology	Biopsy < AIN 2		Biopsy ≥ AIN 2		
	Cytology < HSIL	Cytology HSIL or ASC-H	Cytology < HSIL	Cytology HSIL or ASC-H	
**Cytology <HSIL**	130	9	12	14	165 (63%)
**Cytology HSIL or ASC-H**	47	13	10	26	96 (37%)
	177 (68%)	22 (8.5%)	22 (8.5%)	40 (15%)	261 (100%)

HRA: high resolution anoscopy; AIN: anal intraepithelial neoplasia; HSIL: high grade squamous intraepithelial lesion; ASC-H: atypical squamous cells, can't rule out high grade.

**Figure 1:**
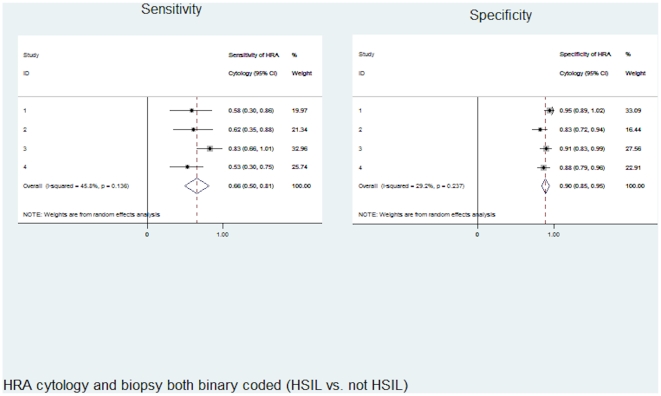
Sensitivity and specificity of HRA cytology for HSIL on biopsy (n = 261 exams), by anoscopist and pooled overall.

Figure 1 presents the unadjusted sensitivity and specificity of HRA cytology for HSIL on HRA-directed biopsy, separately by anoscopist and then pooled over anoscopists. There was no statistical evidence of heterogeneity of effect across anoscopists for either sensitivity or specificity (I-squared p-values 0.136 and 0.237, respectively). The pooled sensitivity (95% CI) and specificity (95% CI) of binary coded HRA cytology (HSIL vs. not HSIL) were 0.66 (0.50–0.81) and 0.90 (0.85–0.95), respectively.

The first adjustment of HRA cytology sensitivity and specificity involves an extrapolation from the findings of Byrom et al.[9] regarding the sensitivity and specificity of colposcopically directed cervical punch biopsy in the detection of HSIL by the LLETZ procedure (taken as a gold standard). Assuming that the sensitivity and specificity of HRA directed anal biopsy for true HSIL is the same as reported by Byrom (74% and 91%, respectively) and also assuming the conditional independence of HRA cytology and HRA biopsy given true disease status, we found the corrected sensitivity and specificity of HRA cytology to be 89% and 96%, respectively (Table 2).

**Table 2:** Unadjusted and adjusted estimates of sensitivity, specificity, LRP_§_, and LRN_§§_ of HRA cytology for HSIL on HRA directed biopsy (n = 261 patients).

	Unadjusted	Adjusted			
		1	2	3	3′
**Sensitivity**	0.66	0.89	0.66	0.74	0.47
**Specificity**	0.90	0.96	0.89	1.0	1.0
* **LRP**	6.6	22.25	6.0	∞	∞
† **LRN**	0.38	0.12	0.38	0.26	0.53

*LRP: likelihood ratio positive [sensitivity/(1– specificity)].

†LRN: likelihood ratio negative [(1– sensitivity)/specificity].

1. Adjusted assuming: (1) sensitivity and specificity of biopsy for HSIL are 0.74 and 0.91, respectively (from Byrom et al.[9]) and (2) conditional independence between cytology and biopsy given true disease status.

2. Adjusted assuming that HRA-directed biopsy has 100% specificity but unknown sensitivity. Then the sensitivity of HRA cytology is estimated without bias as the observed sensitivity (0.66) and the specificity of HRA cytology is estimated by solving equation 19 ( 
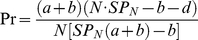
 } of Staquet et al.[11] for specificity. Here we assumed the observed HSIL prevalence of 24% by biopsy.

3. Adjusted using latent class analysis (LCA) assuming conditional independence but imposing restriction that HRA biopsy result of HSIL is measured without error (no false positives).

3′. Adjusted using latent class analysis (LCA) relaxing conditional independence assumption and imposing restriction that HRA biopsy result of HSIL is measured without error (no false positives).

The second adjustment of HRA cytology sensitivity and specificity assumes that HRA biopsy yields pathognomonic results (no false positives). If the observed prevalence of HSIL disease by biopsy (24%) reflects the true prevalence, then the adjusted estimates for HRA cytology sensitivity and specificity are 0.66 (same as the unadjusted sensitivity) and 0.89, respectively (Table 2). As the true prevalence of HSIL disease varies from 24% to 37%, the specificity of HRA cytology varies from 0.89 to 1.00, illustrating the findings of Staquet et al. regarding the dependence of test specificity on disease prevalence when the reference test (HRA biopsy) is assumed to have 100% specificity.

The third adjustment utilizes latent class analysis to estimate HRA cytology sensitivity and specificity from a probabilistic model including the joint binary distributions of the 3 indicator variables (referral cytology, HRA cytology, and HRA biopsy) and imposing the assumption that when the HRA biopsy is read as HSIL, the true disease state is considered to be known (no false positives). Results from two models are presented in Table 2 (labeled 3 and 3′). The first LCA model (column 3) assumed conditional independence and resulted in HRA cytology sensitivity and specificity estimates of 0.74 and 1.0, respectively. In contrast, after relaxing the conditional independence assumption (column 3′), the estimate of HRA cytology sensitivity dropped to 0.47. Review of the classification tables for models 3 and 3′ reveals that the only difference in LCA cluster (latent disease status) assignment between the two models is for the combination of test results: referral cytology “HSIL or ASC-H”, HRA cytology “< HSIL”, and HRA biopsy “<HSIL” (n = 47 out of 261). Model 3 (conditional independence assumed) assigned cases with this result combination to a true disease status of “<HSIL”, whereas model 3′ (relaxed conditional independence assumption) assigned the same cases to the “HSIL” category. The corresponding LCA estimates for true HSIL disease prevalence are 32% [84/261] (model 3) and 50% [131/261](model 3′), contrasted with the observed HSIL biopsy prevalence of 24% that is assumed to be accurate when punch biopsy is considered to be a true gold standard.

**Figure 2:**
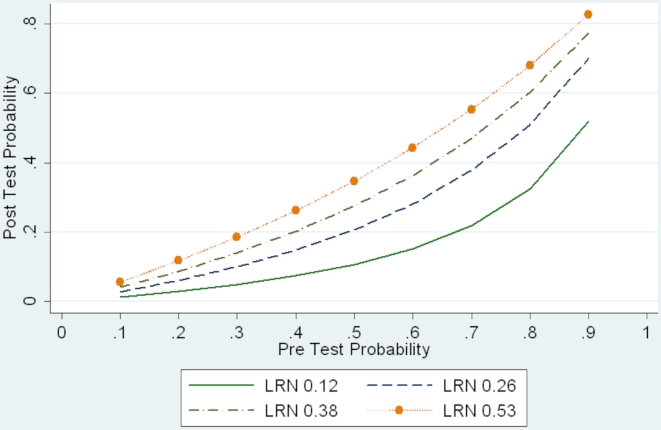
Post test and pretest probabilities of anal HSIL for non-HSIL cytology result, by cytology negative likelihood ratios (LRN).

Finally, Figure 2 presents a sensitivity analysis of the impact of the varying estimates of HRA cytology operating characteristics (shown in Table 2) on the post test probability of true anal HSIL changes after a single non-HSIL cytology result. The figure expresses the cytology operating characteristics as a negative likelihood ratio (defined as [1– sensitivity/specificity]), which is interpretable as a measure of how much *less likely* someone with true HSIL is to have a non-HSIL cytology compared to someone without HSIL tissue changes. As an example, if the true prevalence of anal HSIL in the studied population was 20%, the post test probability of HSIL changes after a non-HSIL cytology varies from 2.9% (LRN  =  0.12) to 11.7% (LRN  =  0.53).

## Discussion

Depending upon what assumptions are made regarding the sensitivity and specificity of HRA directed punch biopsy for true anal HSIL, we found that the estimated sensitivity of anal cytology could range from 47% to 89%. The width of this range (42) based on analysis of the *same dataset* is wider than the range of 24 noted by Chiao et al.[1] in their summary of unadjusted anal cytology operating characteristics from *6 different datasets* derived from studies using similar methodologies and highlights the importance of understanding the accuracy of the reference standard (HRA directed punch biopsy) in evaluating the accuracy of a screening test (anal cytology).

Implications for screening policy of our adjusted analyses of anal cytology operating characteristics are suggested in Figure 2 if one asks the question “Is the post-test probability of true high grade anal dysplasia sufficiently low after a non-HSIL anal cytology that high resolution anoscopy can be deferred?” An evidence based answer would require information on several factors: (1) the prevalence of true HSIL in the population under care; (2)the cytology operating characteristics; (3) the accuracy of HRA diagnosis of high grade disease; (4) the frequency of cytology screening; (5) the rate of disease progression between screenings; and (6) the availability and effectiveness of ablative treatments for HSIL in reducing risk of invasive anal cancer. Figure 2 illustrates that the variation (attributable to assumptions regarding reference standard accuracy) in estimated probability of missing true HSIL changes is smallest at low disease prevalence and increases through plausible estimates of disease prevalence. Unbiased estimates of HSIL prevalence among HIV infected populations are difficult to obtain. Palefsky et al. reported a 4-year incidence of AIN 2 or AIN 3 of 38% in an observational cohort of HIV-infected MSM recruited in the pre-HAART era in San Francisco [15]. Anderson et al. reported a prevalence of anal HSIL of 13% in a clinic based cohort of HIV-infected patients (98% male) with entry CD4 >300 cells/µL [16]. The prevalence of anal HSIL among MSM without HIV infection has been estimated to be approximately 5% [17]. In an unselected population of HIV-infected patients (92% male and 68% MSM) under care, we found a cytologic prevalence of HSIL of 7.5% at initial screening cytology [2]. So the plausible range of cytologic HSIL prevalence among HIV infected male populations may vary from 5% to 38%, varying by risk factors and degree of immunosuppression. Especially toward the higher end of this range, the estimates in Figure 2 would suggest considerable uncertainty regarding how many true HSIL cases would be undiagnosed by deferring HRA among patients with non-HSIL screening cytology.

The assumption involved in extrapolation of the operating characteristics of cervical punch biopsy to anal punch biopsy is more properly considered a hypothesis based on: (1) the common viral pathogenesis of cervical and anal dysplasia [18]; (2) the homologous nature of the cervical and anal transformation zones; (3) comparable diagnostic categories for both cervical and anal dysplasia (modified Bethesda system for CIN and AIN) [7]; (4) the comparability of screening procedures [1]; and (5) the comparability of colposcopic and HRA visual indications of underlying dysplasia [19]. An important difference between the anatomy of the anal canal and of the uterine cervix is the cylindrical collapsing nature of the anal canal, a characteristic which limits full visualization under magnified examination and may reduce the sensitivity of anal punch biopsy in comparison to that of cervical punch biopsy.

Which of our estimates of the sensitivity and specificity of anal cytology are best? The frank answer is that we don't know. However, we lean toward the adjusted estimate of Model 1 (Table 2) that is based on extrapolation from the measured sensitivity and specificity of cervical punch biopsy and on the assumption of conditional independence of cytology and biopsy results given true disease status. Unfortunately, neither of the assumptions involved in this estimate are directly verifiable. But the estimates from Model 1 (sensitivity 89%, specificity 96%) are similar to those derived from Model 3 (sensitivity 74% and specificity 100%), the latent class model assuming conditional independence and also that HRA biopsy result had no false positives. The limitations of the application of latent class analysis to diagnostic test evaluation have been recently reviewed [12], [20] and include the following challenges: (1) the presence or absence of the disease being studied (in this case anal dysplasia) is defined statistically, not clinically; (2) the assumptions of the method are often not directly testable. An advantage of LCA is that it allows use of multiple sources of information in defining disease status, and this is conceptually appealing in our application where screening both by cytology and HRA are typically repeated at varying intervals and the replications can provide additional information regarding true disease status. However, not all preceding replicate tests are of equal value. In previous work [2], we reported that agreement between sequential anal cytology results decreased as the interval between measurements increased, a phenomenon that probably reflects biological evolution over time. Restricting eligibility of referral cytology for inclusion in the analysis to an interval of three to six months from HRA would strengthen the association between referral cytology and HRA measurements but at the cost of reduced sample size and selection bias as those with longer intervals between measurements tend to be those with lower degrees of dysplasia on referral cytology.

In a recent review of anal cytology screening, Ho and Cranston expressed the opinion that the “poor specificity limits the utility of anal cytology as a screening tool given that many patients without high-grade dysplasia will be referred for HRA, resulting in increased healthcare delivery cost and excessive use of clinical resources” [21]. However, this inference would seem to be based on the assumption that the HRA directed punch biopsy represents a true gold standard and that, consequently, a high grade cytology matched with a less than high grade biopsy represents a false positive cytology. We believe that the wide reported ranges of sensitivity and specificity for anal cytology are likely to be attributable to variations in operator performance as well as to extent of disease sampled by both cytology and biopsy. We previously reported a positive association between number of HRA procedures performed and kappa agreement between cytology and biopsy [3]. Regarding disease burden, Nathan et al. recently demonstrated the dependency of anal cytology operating characteristics upon the extent of disease noted at high resolution anoscopy [22].

Our analyses are subject to a number of limitations. First, they are based on observational data subject to selection bias. Consistent with published recommendations [23], not all patients who underwent screening cytology were referred for HRA. In particular there was under representation of patients with no abnormalities on screening cytology. Second, our choice of cytology result cut point (HSIL vs. not HSIL) for the primary analyses of cytology sensitivity and specificity allowed examination of only one point on the ROC curve that summarizes the tradeoff between sensitivity and specificity as the cytology cut point is varied. The overall ROC area estimated for anal cytology in this study (0.78) suggests similar discriminatory ability to comparable estimates of the accuracy of cervical cytology (ROC areas calculated from 2 publications with analyzable data 0.79 [24] and 0.94 [25]). Third, in modeling the corrected operating characteristics of anal cytology we accepted the pathologist's clinical report as itself reliable. However, this approach neglects important variability within and among pathologists in reading the same cytology and biopsy specimens [26]. As Lytwyn et al. have demonstrated, the kappa agreement among four independently reading pathologists with a consensus diagnosis distinguishing HSIL from non-HSIL varied from 0.55 to 0.88 for anal cytology and from 0.76 to 0.94 for anal punch biopsy specimens [26]. Fourth, as latent class analysis requires a minimum of three estimators of the value of the latent class variable, we used the referral cytology as the third estimator in addition to concurrently obtained HRA cytology and HRA punch biopsy. However, only 64% of those who underwent HRA biopsy had both referral and HRA cytology available for analysis.

In conclusion, in advancing the evidence base of screening for anal cancer and its precursors among patient populations at increased risk, it is important to characterize the operating characteristics of each screening component. The measurement of the accuracy of the first component of screening, anal cytological examination, should take into account the fact that the most commonly available reference test, HRA-directed punch biopsy, is itself subject to sampling and measurement error. While there is no single adjustment that unequivocally solves the problem, our analyses under varying assumptions illustrate the range of uncertainty associated with empirical estimates of cytology sensitivity, specificity, and predictive values. Screening policy recommendations should take into account that both anal cytology and HRA evaluation with biopsy provide valuable but fallible complementary information regarding the true extent of dysplasia in the anal canal.

## Supporting Information

Appendix S1 Appendix that describes the derivation of the specificity of a new test as a function of disease prevalence and the joint distribution of observed values of a new test and a reference test.
(0.04 MB DOC)
