# Supplementary material for: Estimating the Accuracy of Anal Cytology in the Presence of an Imperfect Reference Standard
Source: PLoS One. 2010 Aug 19;5(8):e12284. doi: 10.1371/journal.pone.0012284 (PMC2924391; doi:10.1371/journal.pone.0012284)
Supplement: Appendix S1 — Appendix that describes the derivation of the specificity of a new test as a function of disease prevalence and the joint distribution of observed values of a new test and a reference test. (0.04 MB DOC) [file pone.0012284.s001.doc]

Appendix

1. Staquet’s derivation of corrected sensitivity and specificity of a new test when assessed by a reference test with known sensitivity and specificity is given according to the following data structure and equations.

|  | Reference test (R)  + - | |  |
| --- | --- | --- | --- |
| +  New Test (N) - | a | c |  |
|  | b | d |  |
|  |  |  | N |

and , $SP_{N}=\frac{(b+d)S_{R}-b}{NS_{R}-(a+b)}$,

where *S_N_* and *SP_N_* are the sensitivity and specificity of the new test and *S_R_* and *SP_R_* are the corresponding quantities for the reference test. These equations are given as equations (8) and (9) in Staquet’s publication, respectively.

2. For the scenario involving evaluation of a new test against a reference test with 100% specificity and unknown sensitivity, Staquet derived the following formula that expresses disease prevalence as a function of specificity of the new test.

$\Pr=\frac{(a+b)(N\cdot SP_{N}-b-d)}{N[SP_{N}(a+b)-b]}$,

where Pr = Prevalence, *SP_N_* is the specificity of the new test, and the other variables correspond to the data layout above. This is Staquet’s equation (19). When solved for *SP_N_*, this equation allows estimation of the specificity of a new test according to a range of disease prevalence values.

$$SP_{N}=\frac{-ab-ad-b^{2}-bd+N\cdot\Pr\cdot b}{N(a+b)(\Pr-1)}$$
